# Whole Mitochondrial Genome Sequencing and Phylogenetic Tree Construction for *Procypris mera* (Lin 1933)

**DOI:** 10.3390/ani14182672

**Published:** 2024-09-13

**Authors:** Zhe Li, Yaoquan Han, Yusen Li, Weijun Wu, Jianjun Lei, Dapeng Wang, Yong Lin, Xiaoqing Wang

**Affiliations:** 1 College of Fisheries, Hunan Agricultural University, Changsha 410128, China; lzyzy0515@163.com; 2 ASEAN Key Laboratory of Comprehensive Exploitation and Utilization of Aquatic Germplasm Resources, Ministry of Agriculture and Rural Affairs, Key Laboratory of Aquaculture Genetic and Breeding and Healthy Aquaculture of Guangxi, Guangxi Academy of Fishery Sciences, Nanning 530021, China; hyqao@sohu.com (Y.H.);

**Keywords:** Cyprininae, mitochondrial genome, phylogenetic relationships, *Procypris mera*

## Abstract

**Simple Summary:**

As a fish under second-class protection in China, the structure and characteristics of the mitochondrial genome *Procypris mera* (Lin, 1933) have not been reported. We reported three mitochondrial genomes of *P. mera* from three sites and conducted a detailed analysis of their characteristics, which were employed to infer phylogenetic relationships. These findings revealed that the mitochondrial genomes of *P. mera* from the different sites exhibited considerable similarity. Furthermore, the phylogenetic tree constructed from amino acid sequences supported the original phylogenetic relationships of the subfamily Cyprininae in China, suggesting that the genus *Puntioplites* is sister to all other genera of the subfamily Cyprinidae of China; the genus *Procypris* forms a monophyletic group; and the genera *Carassioides*, *Carassius*, and *Cyprinus* form a monophyletic group.

**Abstract:**

*Procypris mera* (Lin, 1933), also known as the Chinese ink carp, currently has a second-class protection status in China. Understanding the structure and characteristics of mitochondrial genes provides essential information for resource conservation and phylogenetic studies of *P. mera*. Here, we sequenced the mitochondrial genomes of three *P. mera* (WYL1-3) from three sites and performed phylogenetic analysis. The generated three genomes were 16,587 bp in length, comprising 13 protein-coding genes (PCGs), 22 tRNAs, two rRNAs, and two non-coding regions (control region (CR), D-loop, and light-stranded replication start OL), with a preference for codons ending in A or C. The mitochondrial genomes of WYL2 and WYL3 were identical, differing from that of WYL1 by only five single-nucleotide polymorphisms (SNPs). All mitochondrial PCGs had Ka/Ks ratios of less than one, suggesting purifying selection. Phylogenetic tree analysis based on amino acid sequences suggested that the genus *Puntioplites* is sister to all other genera of the subfamily Cyprinidae of China; the genus *Procypris* forms a monophyletic group; and the genera *Carassioides*, *Carassius*, and *Cyprinus* form a monophyletic group. This study contributes to our understanding of the phylogenetic relationships in subfamily Cyprininae in China and lays the foundation for resource conservation and management of *P. mera*.

## 1. Introduction

*Procypris mera* (Lin, 1933), also known as the Chinese ink carp, is an endemic species in China and belongs to the order Cypriniformes, family Cyprinidae, subfamily Cyprininae, and genus *Procypris* (S.Y. Lin, 1933) [1]. Historically, *P. mera* was distributed in the upper reaches of the Xijiang River, both in main streams and tributaries [1]. However, over the past two decades, its population has declined, with no resources in many waters, leading to its classification as an endangered species. It is listed as endangered in the Red Book of China’s Endangered Animals and as vulnerable on the IUCN Red List of Threatened Species [2]. Additionally, in 2021, *P. mera* was included in the second tier of China’s State Key Wildlife for Protection; this means that it is a very important protected animal, which is less numerous and is threatened with extinction in the wild. The Guangxi Academy of Fisheries Sciences has been working on the conservation of the *P. mera* resource since 2016. It has cumulatively stocked and released more than 600,000 individuals of *P. mera*. Limited studies have been conducted on its artificial propagation [3], embryonic development [4], and morphological traits [5]. Despite efforts to conserve this species, significant challenges remain. The unclear distribution pattern of *P. mera* is a major obstacle to the development of precise strategies for stock enhancement and effective conservation measures. In addition, there is a lack of valid reference data for the assessment of the genetic diversity of populations.

Mitochondrial DNA (mtDNA) is crucial for genetic studies, with the advantages of rapid evolution, maternal inheritance, small genome size for efficient sequencing and analysis, high copy number (4–6 per mitochondrion across tissues), evolutional stability with limited recombination, and low tissue-specific variation. mtDNA is well conserved and widely used to explore evolutionary origins, construct phylogenetic trees, and analyze population dynamics [6,7,8,9]. Mitochondrial genomes have been extensively used to investigate evolutionary origins, construct phylogenetic trees, develop environmental DNA-specific primers, and analyze population phylogeography [10,11,12].

Therefore, we aimed to sequence the mitochondrial genomes of three *P. mera* individuals from different sites to determine whether there are differences between populations and to construct a phylogenetic tree for the subfamily Cyprininae in China. The objective of this study was to provide basic data for the further development of environmental DNA-specific primers and analysis of genetic diversity in *P. mera*, and to lay the foundation for conserving and managing *P. mera* resources.

## 2. Materials and Methods

### 2.1. Sample Collection and DNA Extraction

**Figure 1:**
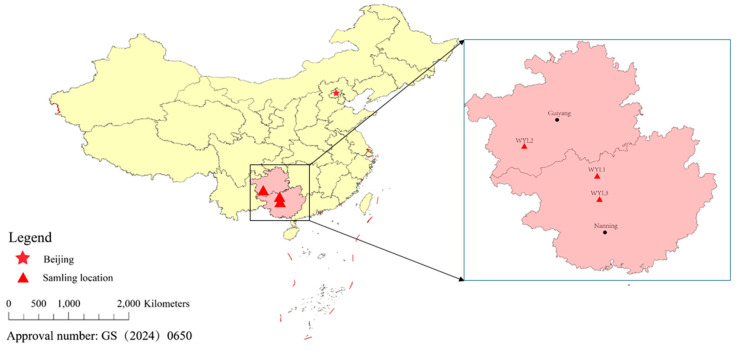
The sampling locations of *P. mera*.

Three individuals of *P. mera* were collected from three different locations, including Jinchengjiang District, Hechi City, Guangxi Zhuang Autonomous Region, China (WYL1); Zhenfeng County, Qianxi’nan Prefecture, Guizhou Province, China (WYL2); and Du’an Yao Autonomous County, Hechi City, Guangxi Zhuang Autonomous Region, China (WLY3) (Figure 1). Genomic DNA was isolated from the pectoral fin using the phenol-chloroform method, and the quality and integrity of DNA samples were measured using 0.8% agarose electrophoresis, Nanodrop spectrophotometer (Thermo Fisher Scientific, Waltham, MA, USA), and Qubit^®^ 2.0 Flurometer (Life Technologies, Foster City, CA, USA).

### 2.2. Mitochondrial Genome Sequencing and Assembly

The obtained DNA samples were used to construct a double-ended sequencing library with an insert size of 350 bp, per the standard procedure for Illumina DNA library construction. The quality of the library was assessed using qPCR method and the Agilent 2100 Bioanalyzer (Agilent Technologies, Palo Alto, CA, USA). Qualified DNA libraries were sequenced on an Illumina Hiseq 4000 (Illumina, San Diego, CA, USA) high-throughput sequencing platform using a PE150 (Pair-End 150) sequencing strategy. The raw sequences were filtered to eliminate those containing sequencing junctions and those of low quality, ensuring high-quality clean reads for subsequent analysis. Mitochondrial genome sequences were assembled via splicing using the SPAdes v.3.5.0 software (https://github.com/ablab/spades, accessed on 20 July 2024) [13].

### 2.3. Mitochondrial Genome Annotation and Analyses

The mitochondrial genome was annotated using DOGMA (http://dogma.ccbb.utexas.edu/ (accessed on 10 May 2018)) [14] and ORF Finder (https://www.ncbi.nlm.nih.gov/orffinder/ (accessed on 15 May 2018)). The preliminary results of the annotation were compared with the reported coding proteins and rRNAs of the mitochondrial genomes of related species using BLASTn and BLASTp (https://blast.ncbi.nlm.nih.gov/Blast.cgi (accessed on 17 May 2018)) to verify the accuracy of the results and correct them where necessary [15]. tRNA annotation was conducted using tRNAscan-SE2.0 (http://lowelab.ucsc.edu/tRNAscan-SE/ (accessed on 20 May 2018)) [16] and ARWEN (Version 1.2, http://mbioserv2.mbioekol.lu.se/ARWEN/ (accessed on 25 April 2018)) [17] methods to eliminate tRNAs with implausible length and incomplete structure. We predicted the structure of tRNAs using the online website https://lowelab.ucsc.edu/tRNAscan-SE/ (accessed on 20 July 2024) [16] and mapped the tRNA structure using the online website cloud.genepioneer.com (accessed on 21 July 2024).

The relative synonymous codon usage (RSCU) was calculated according to the formula detailed in Sharp PM [18]. The mitochondrial genome skew values were calculated using the following formula: AT-skew = (A − T)/(A + T); GC-skew = (G − C)/(G + C). Comparative analyses of the genome-wide differences in the mitochondrial genomes were performed using MEGA11 [19].

The best-matching proteins were obtained using BLASTp (parameters -evalue 1 × 10^−5^ -max_target_seqs) [15]. The protein sequence comparison results were converted into CDS sequence comparison results using ParaAT (parameters -m mafft -f axt -g -k) [20], and the Ka/Ks values were calculated using KaKs_Calculator2.0 [21]. A Ka/Ks frequency distribu-tion plot was generated using the website https://www.omicshare.com/tools/ (accessed on 15 July 2024) [22].

### 2.4. Phlogenetic Analyses

To understand the evolutionary status of *P. mera*, we constructed phylogenetic trees of the subfamily Cyprininae in China. The mitochondrial genomes of 16 fish were obtained from the National Center for Biotechnology Information (NCBI) (Table 1). A phylogenetic tree of these mtDNA sequences was constructed using codon and amino acid sequences based on maximum likelihood (ML) and Bayesian methods, respectively. The ML tree was constructed using the IQTree2 for codon and amino acid sequences of 20 mitochondrial genomes, respectively [23]. The sequences were converted to a phylogenetic format following a comparison based on MAFFT software (Version 7.526, --localpair --maxiterate 1000) [24], and the optimal model was selected using IQTree2 based on the AIC value. The optimal substitution model for amino acid sequences was identified as (mtVer + F + R2), whereas the optimal substitution model for codons was identified as (GTR + F + G4). Afterward, the phylogenetic trees for amino acids and codons were constructed according to the optimal model with a bootstrap value of 1000. To determine the optimal topology, a Bayesian tree was constructed using Phylosuite software (Version 1.2.3) [25], beginning with a random tree. The Markov chain was then run for 1,000,000 generations with samples taken at 100-generation intervals. The initial 25% of the samples were excluded, owing to their advanced age, following confirmation that the mean standard deviation of the split frequencies was less than 0.01. A genome covariance map of 20 mitochondrial genomes was constructed using the Mauve software (https://darlinglab.org/mauve/download.html, accessed on 15 July 2024) [26].

## 3. Results

### 3.1. Mitochondrial Genome Analyses

The mitochondrial genome sequencing data for the three *P. mera* individuals are shown in Table 2. The total length of each *P. mera* mitochondrial genome was 16,587 bp, with a GC content of 43.1%, and the distribution of its bases is shown in Table 3. The three mitochondrial genome structures were consistent, consisting of 37 typical animal mitochondrial genes, including 22 tRNA genes, 13 PCG genes, 2 rRNA genes, and 2 non-coding regions (D-loop and OL), which are similar to those of other vertebrates. Among the mitochondrial genes, nine genes (tRNA-Gln, tRNA-Ala, tRNA-Asn, tRNA-Cys, tRNA-Tyr, tRNA-Ser, ND6, tRNA-Glu, and tRNA-Pro) are encoded by the light (L) strand, whereas the remaining genes are encoded by the heavy (H) strand (Table 4).

**Figure 2:**
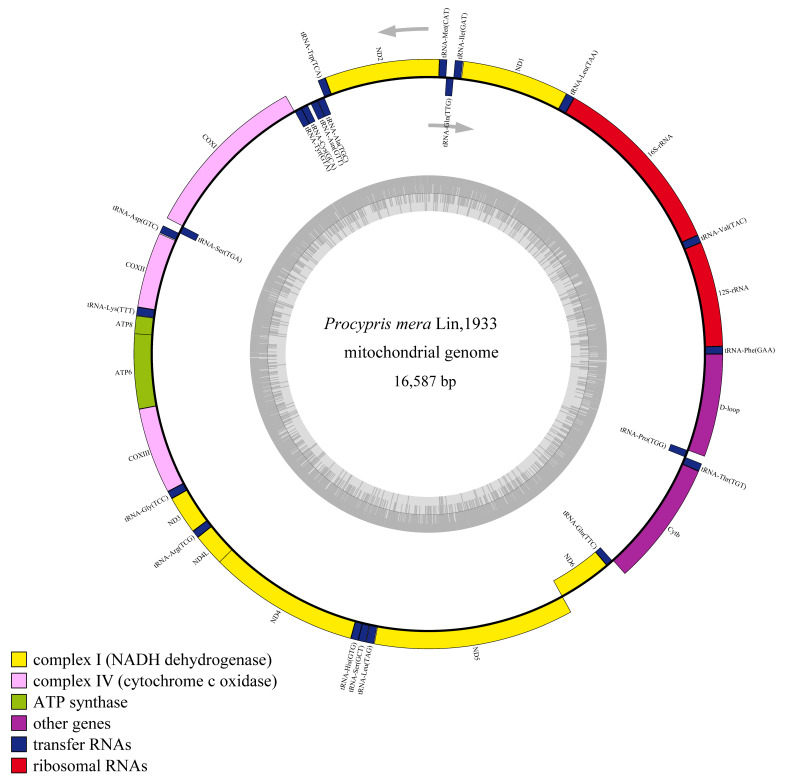
Mitochondrial genome map of the *P. mera*.

Differential analysis of the three mitochondrial genomes revealed that the mitochondrial genomes of WYL2 and WYL3 were identical, whereas WYL1 exhibited five single-nucleotide polymorphisms (SNPs) that distinguished it from the other two (Table 5). One of the five SNPs was identified in the 16S rRNA gene, whereas the remaining four were located in the coding genes COX2, ATP6, ND5, and CYTB. The SNP in ND5 resulted in an amino acid change (Thr to Ala), whereas the other three SNPs were synonymous (Table 5). The mtDNAs of WYL1 and WYL2 were used for subsequent analyses. The mitochondrial structures of WYL1 are shown in Figure 2.

### 3.2. Protein Coding Gene Analyses

The total length of the 13 PCGs in the two mitochondrial genomes was 11,403 bp, with AT and GC skew values of 0.0490 and 0.2848, respectively. Except for COX1, all the PCGs used the ATG start codon. Among the PCGs, seven utilized the complete stop codon TAA, whereas six used incomplete stop codons (TA or T) (Table 4). Furthermore, we identified four overlapping regions among certain PCGs (ATPase6-COX3, ATPase8-ATPase6, ND4-ND4L, and ND5-ND6). These overlapping regions spanned a length of 1–7 bp, with the largest region occurring between ATP8-ATP6 and ND4-ND4L.

**Figure 3:**
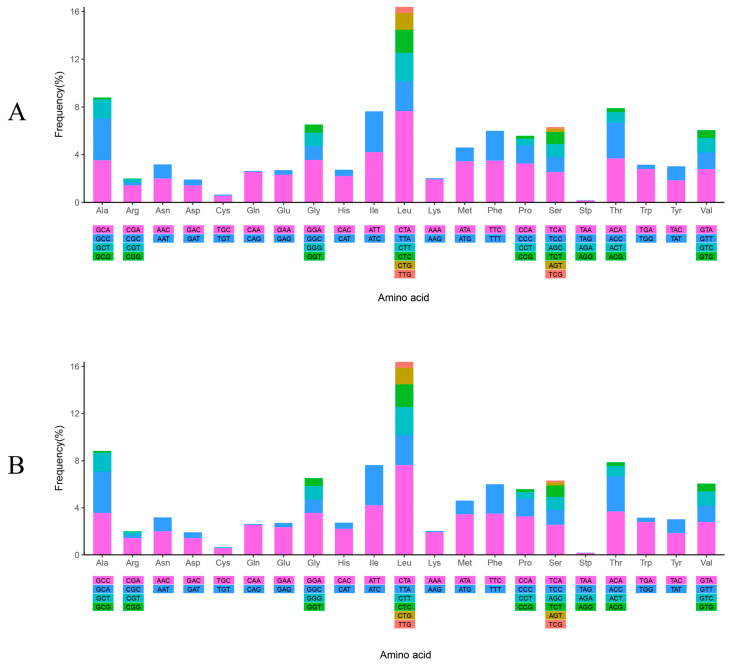
The frequency of relative synonymous codon usage (RSCU) in the mitogenome of *P. mera*WYL1 (**A**) and *P. mera*WYL2 (**B**).

The frequencies and values of RSCU in the two mitochondrial genomes were partially different under the influence of the five SNPs (Figure 3 and Figure S1). Among the codons, CTA was the most frequently utilized, occurring 291 and 290 times in WYL1 and WYL2, respectively. In contrast, TAG was the least frequently utilized, occurring only once in both mtDNAs. In both mtDNAs, there were 26 codons with RSCU values exceeding 1, and 14 codons terminated in A, 11 in C, and 1 in T, indicating a codon preference for those that ended in A and C.

### 3.3. Ribosomal RNA, Transfer RNA Genes, and Non-Coding Regions Analyses

**Figure 4:**
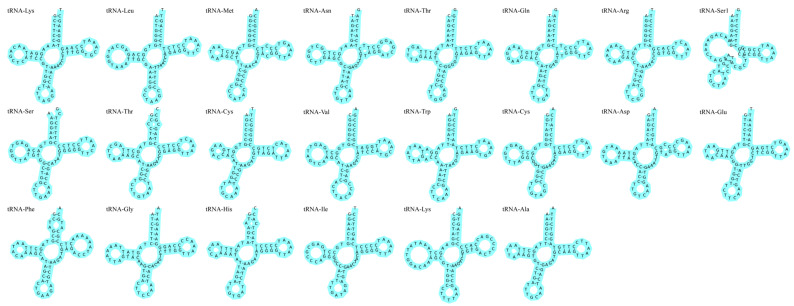
Putative secondary structure of *P. mera* tRNA.

The total length of rRNAs in the two mtDNA samples was 2638 bp. The AT skew value for WYL1 was 0.2895, and the GC skew value was −0.1005. However, the AT skew value for WYL2 was 0.2890, and the GC skew value was −0.0996. There were 22 tRNAs in the mtDNA of *P. mera*, with a total length of 1563 bp in both mtDNA. Three overlapping regions were identified between the tRNAs (tRNA-Cys-tRNA-Tyr, tRNA-lle-tRNA-Gln, and tRNA-Thr-tRNA-Pro). These overlapping regions had a length of 1–2 bp, with the largest region occurring between tRNA-lle-tRNA-Gln. Each tRNA had a length of 67–76 bp, and the secondary structure of all the tRNAs, except for tRNA-Ser1 on the L strand, was of the standard cloverleaf type (Figure 4).

Two common non-coding regions (OL and CR) were identified in the *P. mera* mitogenome. The OL region was 34 bp in length and located between tRNA-Asn and tRNA-Cys. The CR region was located between tRNA-Pro and tRNA-Phe, which is the longest non-coding region in the entire mitochondrial genome, with a length of 938 bp.

### 3.4. Ka/Ks Ratios Analyses

**Figure 5:**
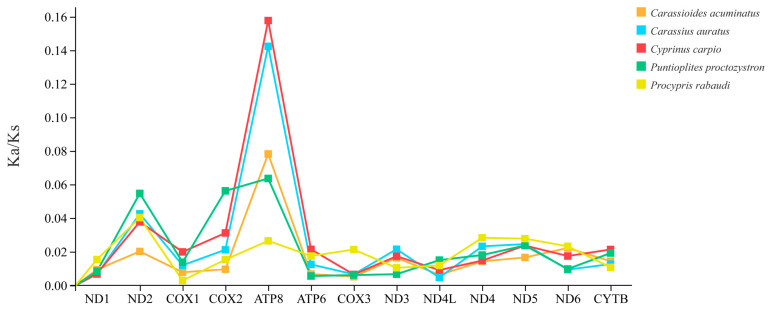
The ratios of Ka/Ks of 13 PCGs of the *P. mera* WYL1.

The WYL1 mtDNA was used to calculate non-synonymous substitution rates (Ka) and synonymous substitution rates (Ks) to assess the selection pressure exerted on the *P. mera* PCGs during evolutionary history. It was shown that the Ka/Ks ratios in all the PCGs ranged from 0.0029 to 0.3411, and the Ka/Ks ratios of each PCG were similar. However, the largest range of variation in Ka/Ks ratios was observed for ATP8, with a range of 0.0265 to 0.3411 (Figure 5). Furthermore, the Ka/Ks ratios of all PCGs were less than one, indicating that purifying selection played a leading role in the evolution of these PCGs.

### 3.5. Phylogenetic Relationships

**Figure 6:**
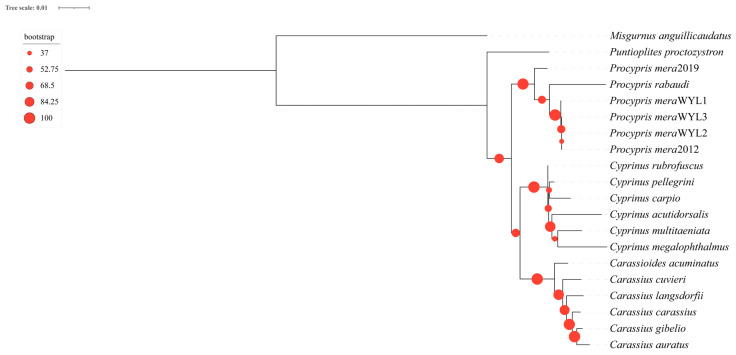
ML tree on the nodes constructed by using the amino acid sequences of the 13 PCGs with bootstrap values.

An analysis of 20 mitochondrial genome sequences from 16 species revealed inconsistencies between phylogenetic trees constructed using codon and amino acid sequences with IQtree2. Specifically, the trees displayed distinct topologies. The amino acid-based phylogenetic tree indicated that the genus *Puntioplites* is sister to all other genera of subfamily Cyprinidae of China. The genus *Procypris* formed a monophyletic group, and the genera *Carassioides*, *Carassius*, and *Cyprinus* formed a monophyletic group (Figure 6). Furthermore, the codon-based phylogenetic tree indicated that the genera *Carassioides* and *Carassius* were monophyletic, as were the genera *Puntioplites*, *Procypris*, and *Cyprinus* a monophyletic group (Figure S2). Validation of the phylogenetic tree using a BI tree resulted in convergence with mean standard deviations of 0.001475 for codon-based and 0.001488 for amino-acid-based sequences, indicating the high reliability of the constructed trees. Results obtained from the BI tree were consistent with the aforementioned findings (Figures S3 and S4). Additionally, a collinearity analysis of the 20 mitochondrial genomes revealed a high degree of conservation in the mitochondrial genomes of fish in the subfamily Cyprininae in China. However, some sequence rearrangements were observed in *Carassius catassius*, *Carassius cuvieri*, *Carassius gibelio*, and *Cyprinus carpio* (Figure S5).

## 4. Discussion

### 4.1. Mitochondrial Differences between Individuals

In this study, we sequenced the mitochondrial genomes of three individuals sourced from three sites, finding that the genomes exhibited remarkable similarity, with the mitochondrial genomes of *P. mera*WYL2 and *P. mera*WYL3 being identical. On the one hand, the striking similarity observed in the mitochondrial genomes can be attributed to the fact that these three individuals likely descended from the same ancestral lineage, consistent with the findings from the mitochondrial genome sequencing of *Salvethymus svetovidovi* [27], a species exclusively and consistently inhabiting Lake El’gygytgyn. On the other hand, the observed low polymorphism could be attributed to the significant population changes that *P. mera* has undergone in recent decades, potentially due to genetic bottleneck effects [28]. However, it is important to note that only one individual from each site was included in this study, which may limit the accuracy of the results. To fully assess the genetic diversity of *P. mera*, additional samples should be collected from each site in the future, and multiple genetic markers should be used for a more robust analysis.

### 4.2. Phylogenetic Relationships of Subfamily Cyprininae

In China, the subfamily Cyprininae includes five genera of fish: *Cyprinus*, *Procypris*, *Puntioplites*, *Carassioides*, and *Carassius* [29]. The subfamily Cyprininae is a monophyletic group Barbelidae [29,30]. To clarify the phylogenetic relationships of the subfamily Cyprininae, researchers have compared the traits of the subfamily Cyprininae with those of the subfamily Barbelidae. Chen et al. [30] divided the genera of the subfamily Cyprininae into three groups based on pharyngeal tooth morphology. Spatulate teeth were found in the genera *Puntioplites* and *Procypris*, molar teeth were observed in the genera *Cyprinus* and *Scaphognathops,* and shovel teeth were found in the genera *Carassioides* and *Carassius* [30]. The genus *Puntioplites* reportedly has spatulate teeth that are generally similar to those of the subfamily Barbelidae, and they are of more primitive types [30]. Zhou [31] conducted a comparative analysis of 25 traits exhibited by fish in the subfamily Cyprininae and those observed in fish in the subfamily Barbus. They concluded that the genus *Procypris* was more closely related to *Cyprinus*, whereas *Puntioplites* represented an early divergent member of the subfamily Cyprininae and was more distantly related to the other four genera within the subfamily [31].

Classification methods based on morphological traits have significantly contributed to the advancement of our understanding of the phylogeny of the subfamily Cyprininae in China, despite the lack of developments in molecular biology at the time. However, it is important to note that morphological variations are not always consistent with molecular evidence [32]. Additionally, the compilation of these traits is often subjective, reflecting the level of attention paid to morphology and taxonomy and the intensity of taxon sampling [32]. Hence, studies on the phylogeny of the subfamily Cyprininae based on molecular evidence will help to reorganize the developmental relationships of the subfamily. The phylogenetic tree constructed by Yue, based on mitochondrial D-loop sequences, indicated that the genus *Procypris* is a sister group to the genera *Carassioides*, *Carassius*, and *Cyprinus* [33]. Nevertheless, the absence of molecular evidence for the genus *Puntioplites* precluded a systematic analysis of the phylogenetic tree of the subfamily Cyprininae [33].

In the present study, we sequenced the mitochondrial genome of *P. mera* and constructed a phylogenetic tree that included 15 fish of the subfamily Cyprininae in China. The phylogenetic trees constructed based on codon sequences using both ML and Bayesian methods indicated that the genera *Carassius* and *Carassioides* represent a monophyletic group, whereas the genera *Puntioplites*, *Procypris*, and *Cyprinus* constitute another monophyletic group, which is not consistent with analyses based on morphology [29,30]. Additionally, the amino-acid-based phylogenetic tree indicated that the genus *Puntioplites* is sister to all other genera of the subfamily Cyprinidae of China, and that the genera *Procypris*, *Carassioides*, *Carassius*, and *Cyprinus* formed a monophyletic group. This finding is consistent with numerous conclusions based on molecular evidence and morphological studies [29,30,33,34,35,36].

## 5. Conclusions

Herein, we sequenced three mitochondrial genomes of *P. mera*, analyzed their structural features, and constructed a phylogenetic tree of the subfamily Cyprininae in China based on newly published mitochondrial genomes. Our findings revealed that the three mitochondrial genomes of *P. mera* exhibited identical lengths, with minor variations in SPNs. Structural analysis highlighted conserved features across the genomes, such as ATG start codons in most PCGs and the standard cloverleaf structure of tRNA genes, except for the absence of a DHU stem in trnS1. Additionally, purifying selection was identified as a key evolutionary force shaping the evolution of these PCGs. A phylogenetic tree for the subfamily Cyprininae in China was constructed using codon and amino acid sequences based on ML and Bayesian methods. The results of the phylogenetic tree constructed based on amino acid sequences were similar to those of most previous studies, suggesting that the genus *Puntioplites* is sister to all other genera of subfamily Cyprinidae of China; the genus *Procypris* forms a monophyletic group; and the genera *Carassioides*, *Carassius*, and *Cyprinus* form a monophyletic group. However, an accurate phylogenetic tree for fish of the subfamily Cyprininae still requires whole-genome refinement. In summary, this research significantly advances our understanding of the phylogenetic intricacies within the subfamily Cyprininae in China, thereby establishing a solid foundation for the development of future conservation and management strategies for *P. mera*.

## Figures and Tables

**Table 1:** Taxonomic information and GenBank accession number of all species used in the phylogenetic analysis.

Genus	Species	GenBank Accession Number	Length (bp)
*Carassioides* (Ōshima, 1926)	*Carassioides acuminatus* (J. Richardson, 1846)	KX602324.1	16,579
*Carassius* (Nilsson, 1832)	*Carassius auratus* (Linnaeus, 1758)	EF483931.1	16,581
	*Carassius carassius* (Linnaeus, 1758)	AY714387.1	16,580
	*Carassius cuvieri* (Temminck and Schlegel, 1846)	NC_010768.1	16,581
	*Carassius gibelio* (Bloch, 1782)	GU138989.1	16,582
	*Carassius langsforfi* (Temminck and Schlegel, 1846)	NC_002079.1	16,578
*Cyprinus* (Linnaeus, 1758)	*Cyprinus megalophthalmus* (Wu et al., 1963)	KR869143.1	16,580
	*Cyprinus carpio* (Linnaeus, 1758)	NC_001606.1	16,575
	*Cyprinus multitaeniata* (Pellegrin and Chevey, 1936)	KR869145.1	16,580
	*Cyprinus pellegrini* (Tchang, 1933)	MN718675.1	16,583
	*Cyprinus rubrofuscus* (Lacepede, 1803)	MW969691.1	16,582
	*Cyprinus acutidorsalis* (Chen and Hwang, 1977)	KR869144.1	16,580
*Procypris*	*Procypris mera2019* (Lin, 1933)	MN841761.1	16,579
	*Procypris mera2012* (Lin, 1933)	JX316027.1	16,586
	*Procypris mera*WLY1 (Lin, 1933)	MN229744	16,587
	*Procypris mera*WLY2 (Lin, 1933)	MN229745	16,587
	*Procypris mera*WLY3 (Lin, 1933)	MN229746	16,587
	*Procypris rabaudi* (Tchang, 1930)	EU082030.1	16,595
*Puntioplites* (H. M. Smith, 1929)	*Puntioplites proctozystron* (Bleeker, 1865)	AP011247.1	16,584
*Misgurnus* (Lacépède, 1803)	*Misgurnus anguillicaudatus* (Cantor, 1842)	MF579257.1	16,647

Note: The designation “*Procypris mera*2019” and “*Procypris mera*2012” indicate that the genome was submitted to the NCBI in 2019 and 2012, respectively.

**Table 2:** Mitochondrial genome sequencing data of three *P. mera*.

	Length (bp)	Reads	Base (G)	Q20	Q30	GC (%)
WYL1	150	4,596,214	1.38	96.47	91.95	38.16
WYL2	150	4,288,544	1.29	96.20	91.45	38.16
WYL3	150	3,490,319	1.05	96.23	91.47	38.41

**Table 3:** The characterisation of the mitochondrial genome three *P. mera*.

	A (bp)	T (bp)	G (bp)	C (bp)	Length (bp)	GC (%)	GC-skew (%)	AT-skew (%)
WYL1	5304	4133	2633	4517	16,587	43.1	−0.2635	0.1241
WYL2	5303	4133	2634	4517	16,587	43.1	−0.2633	0.124
WYL3	5303	4133	2634	4517	16,587	43.1	−0.2633	0.124

**Table 4:** Mitochondrial genome content and structure of *P. mera*.

Gene /Element	Location	Length (bp)	Strand	Initiation Codon	Termination Codon	Intergenic Nucleotide (bp)	Gene /Element	Location	Length (bp)	Strand	Initiation Codon	Termination Codon	Intergenic Nucleotide (bp)
tRNA-Phe	1–69	69	H			0	tRNA-Lys	7872–7947	76	H			1
12S-rRNA	70–1024	955	H			0	ATP8	7949–8113	165	H	ATG	TAG	−7
tRNA-Val	1025–1096	72	H			0	ATP6	8107–8790	684	H	ATG	TAA	−1
16S-rRNA	1097–2779	1683	H			0	COXIII	8790–9574	785	H	ATG	TA	0
tRNA-Leu	2780–2855	76	H			1	tRNA-Gly	9575–9646	72	H			0
ND1	2857–3831	975	H	ATG	TAA	4	ND3	9647–9995	349	H	ATG	T	0
tRNA-Ile	3836–3907	72	H			−2	tRNA-Arg	9996–10,065	70	H			0
tRNA-Gln	3906–3976	71	L			1	ND4L	10,066–10,362	297	H	ATG	TAA	−7
tRNA-Met	3978–4046	69	H			0	ND4	10,356–11,736	1381	H	ATG	T	0
ND2	4047–5091	1045	H	ATG	T	0	tRNA-His	11,737–11,805	69	H			0
tRNA-Trp	5092–5162	71	H			2	tRNA-Ser	11,806–11,874	69	H			1
tRNA-Ala	5165–5233	69	L			1	tRNA-Leu	11,876–11,948	73	H			3
tRNA-Asn	5235–5307	73	L			33	ND5	11,952–13,775	1824	H	ATG	TAA	−4
tRNA-Cys	5341–5407	67	L			−1	ND6	13,772–14,293	522	L	ATG	TAA	0
tRNA-Tyr	5407–5477	71	L			1	tRNA-Glu	14,294–14,362	69	L			5
COXI	5479–7029	1551	H	GTG	TAA	0	Cytb	14,368–15,508	1141	H	ATG	T	0
tRNA-Ser	7030–7100	71	L			3	tRNA-Thr	15,509–15,580	72	H			−1
tRNA-Asp	7104–7175	72	H			5	tRNA-Pro	15,580–15,649	70	L			0
COXII	7181–7871	691	H	ATG	T	0	D-loop	15,650–16,587	938	H			0

**Table 5:** The annotation data of SNPs.

Position	WYL2	WYL1	Gene Type	Gene	Codon	Amino Acid	Mutation Type
1296	A	G	rRNA	16S-rRNA			SNP
7354	C	T	CDS	COX2	TCC > TCT	Ser > Ser	SNP
8502	A	G	CDS	ATP6	GAA > GAG	Glu > Glu	SNP
12,774	G	A	CDS	ND5	GCC > ACC	Ala > Thr	SNP
14,503	T	C	CDS	CYTB	TTA > CTA	Leu > Leu	SNP

## Data Availability

All three mitochondrial genomes of *Procypris mera* measured in this study have been uploaded to NCBI (MN229744, MN229745, MN229746).

## Supplementary Materials

The following Supporting Information can be downloaded at: https://www.mdpi.com/article/10.3390/ani14182672/s1, Figure S1. The value of relative synonymous codon usage (RSCU) in the mitogenome of *P. mera* WYL1 (A) and *P. mera* WYL2 (B). Figure S2. ML tree on the nodes constructed by using the codon sequences of the 13 PCGs with bootstrap values. Figure S3. BI tree on the nodes constructed by using the amino acid sequences of the 13 PCGs with support values. Figure S4. BI tree on the nodes constructed by using the codon sequences of the 13 PCGs with support values. Figure S5. The collinearity map of whole mtDNA. Each mitochondrial genome is partitioned into two segments. The upper segment encompasses the collinearity of homologous regions, where identical blocks of color (homology blocks) signify homologous regions. The lower segment constitutes a map of the gene structure, with white rectangles denoting CDS, red rectangles representing rRNAs, and green rectangles indicating tRNAs.
