# Supplementary material for: Whole Mitochondrial Genome Sequencing and Phylogenetic Tree Construction for Procypris mera (Lin 1933)
Source: Animals (Basel). 2024 Sep 13;14(18):2672. doi: 10.3390/ani14182672 (PMC11428242; doi:10.3390/ani14182672)
Supplement: Supplementary file 1 [file animals-14-02672-s001.zip › animals-3161371-supplementary.pdf]

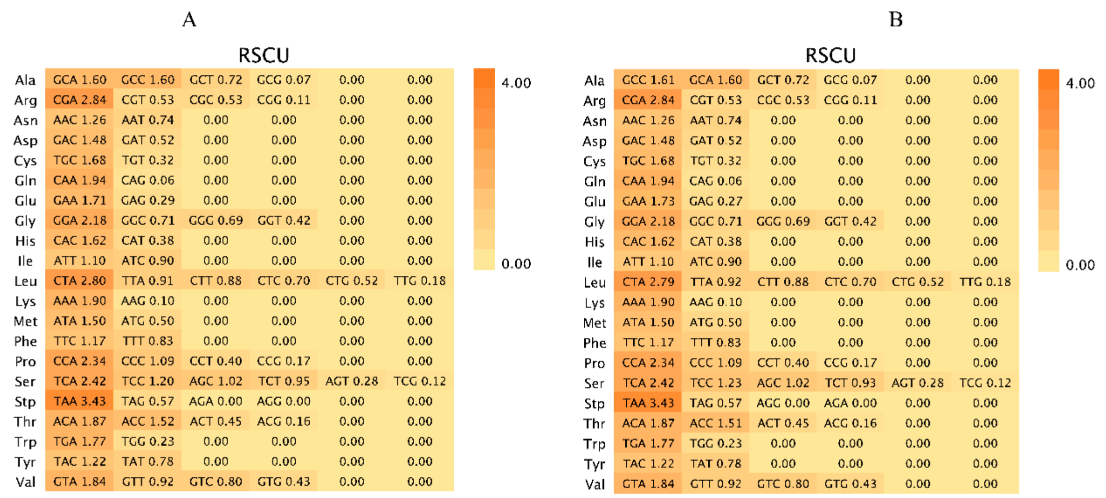

Figure S1. The value of relative synonymous codon usage (RSCU) in the mitogenome of *P.mera*WYL1 (A) and *P.mera*WYL2 (B).

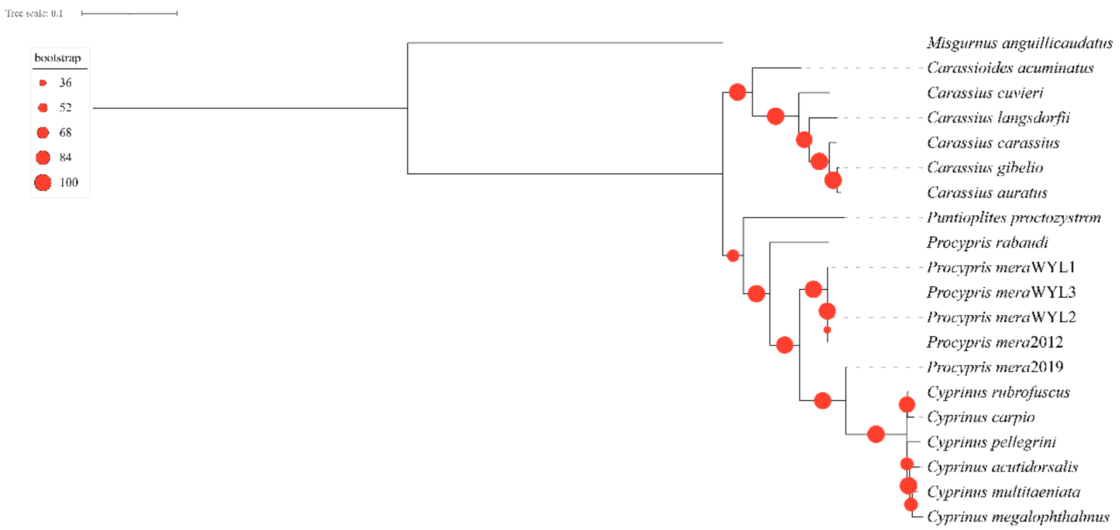

Figure S2. ML tree on the nodes constructed by using the codon sequences of the 13 PCGs with bootstrap values.

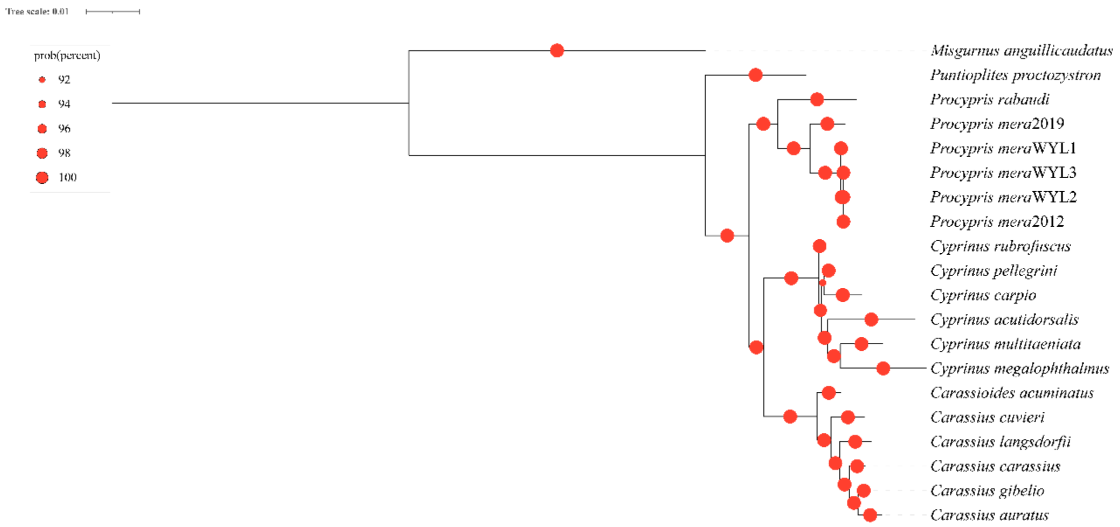

Figure S3. BI tree on the nodes constructed by using the amino acid sequences of the 13 PCGs with support values.

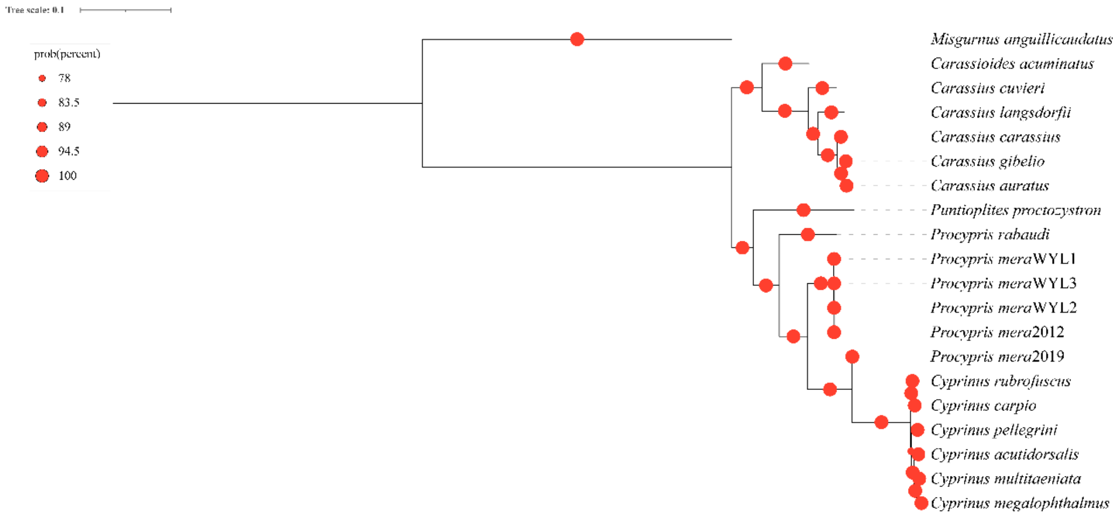

Figure S4. BI tree on the nodes constructed by using the codon sequences of the 13 PCGs with support values.

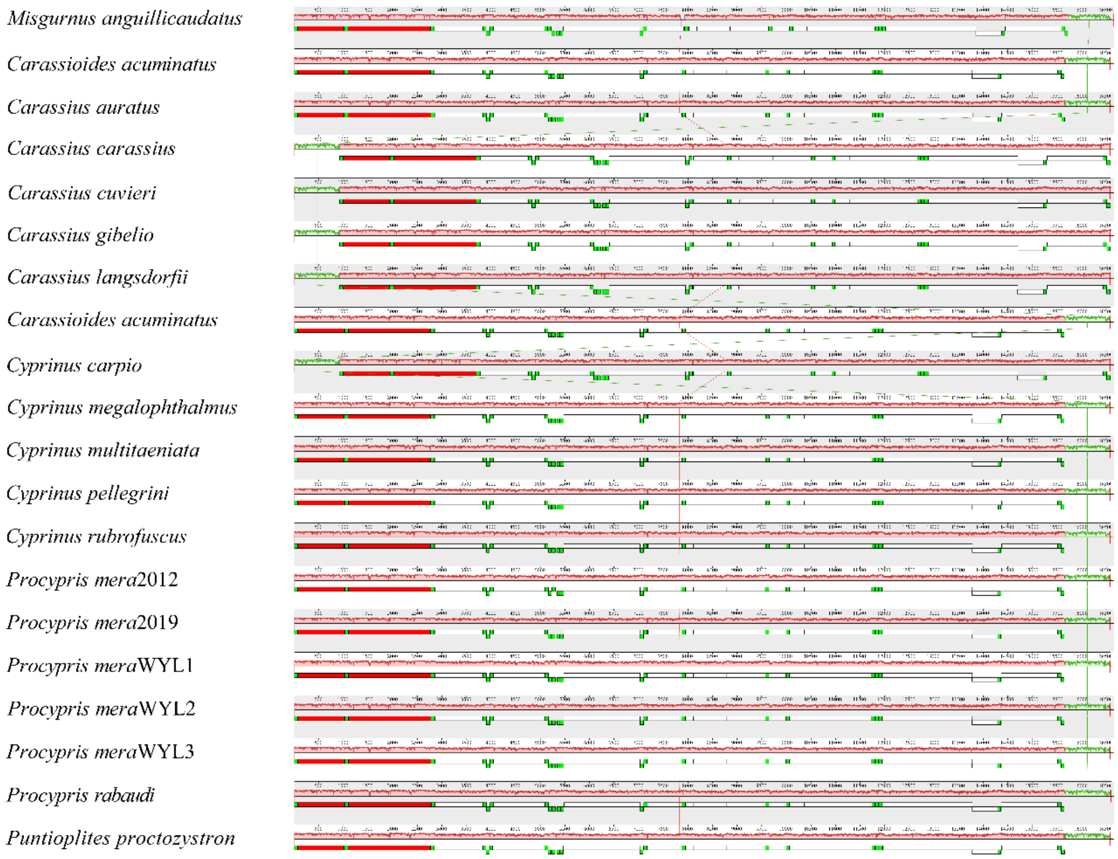

Figure S5. The collinearity map of whole mtDNA. Each mitochondrial genome is partitioned into two segments. The upper segment encompasses the collinearity of homologous regions, where identical blocks of color (homology blocks) signify homologous regions. The lower segment constitutes a map of the gene structure, with white rectangles denoting CDS, red rectangles representing rRNAs, and green rectangles indicating tRNAs.
